# How to find a forgotten disease like yaws: Lessons from the Philippine experience

**DOI:** 10.1371/journal.pntd.0011515

**Published:** 2023-09-14

**Authors:** Belen Lardizabal Dofitas

**Affiliations:** 1 Department of Dermatology, College of Medicine, University of the Philippines Manila-Philippine General Hospital, Metro Manila, Philippines; 2 Department of Public Health, Erasmus MC, University Medical Center Rotterdam, Rotterdam, the Netherlands; UConn Health, UNITED STATES

## Introduction

Yaws is a highly infectious, chronic, and disabling neglected tropical disease of the skin and bones that is caused by *Treponema pallidum* subspecies *pertenue* and mainly affects children in tropical countries. A yaws eradication campaign from the 1950s to the early 1960s was successful in reducing cases by 95% worldwide, but a resurgence was noted in the 1970s. In 2012, the World Health Organization (WHO) targeted eradication of yaws through a mass administration of one-dose azithromycin. Nevertheless, in 2021, a total of 123,866 cases were reported from 13 countries, and 1,102 cases from nine countries were confirmed. Confirmed cases were largely from the Western Pacific region, although most cases from this region were not laboratory confirmed [1].

## Yaws in the Philippines

Yaws has historically occurred in most parts of the Philippines since the Spanish and American colonial times, affecting 10% to 30% of the population in some provinces, especially in the Mindanao island group of the Southern Philippines [2]. After a nationwide yaws eradication campaign in the 1950s, prevalence dropped to 0.4%, and it was generally thought to have been eradicated. After 1973, yaws ceased to be a notifiable disease [3].

**Fig 1:**
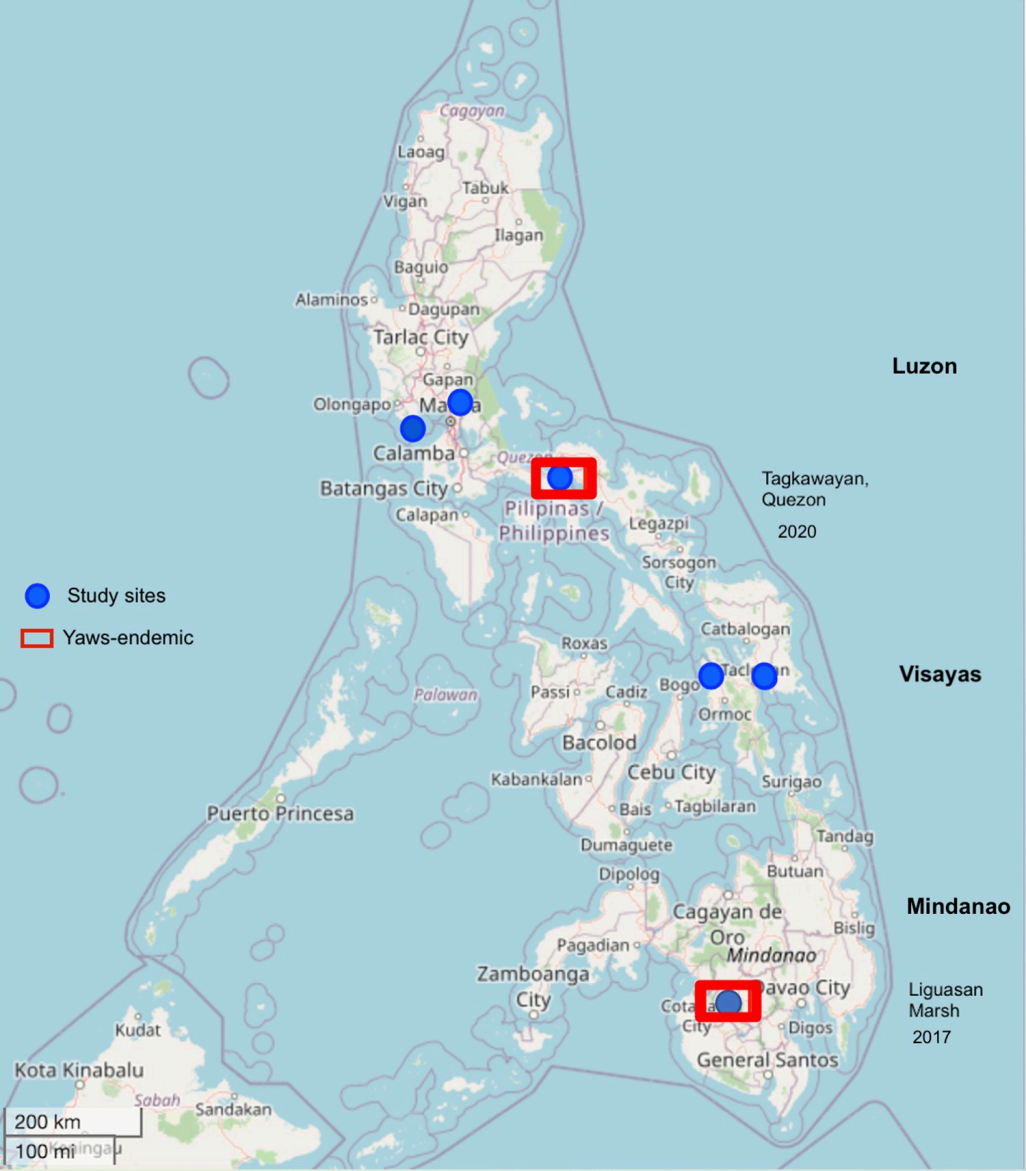
Yaws-endemic areas and study sites in the Philippines. Yaws was confirmed in 2 areas: Liguasan Marsh (2017) and Tagkawayan, Quezon (2020) (contains information from OpenStreetMap and OpenStreetMap Foundation, which is made available under the Open Database License https://www.openstreetmap.org/copyright) [6].

In the year 2000, I first encountered suspected yaws in the Liguasan Marsh of the Mindanao island group and reported it to the national health department. Serologic tests were not performed. In 2017 and 2020, the Philippine Department of Health commissioned me to conduct 2 cross-sectional studies to guide health policy in the development of a national yaws control and eradication program. Only 14 towns and villages across the Philippines were surveyed, yet 19 confirmed active and latent yaws cases were detected at 6 of these study sites [4,5] (Fig 1).

The Philippines once was in the WHO’s Group B epidemiological category, capturing “previously endemic countries, unknown current status.” In 2017, the Philippines was declared the 14th yaws-endemic country and the first Group B country to confirm the presence of yaws since the WHO’s 2012 eradication campaign began. However, at least 76 other previously endemic countries in the Group B category are not yet assessing yaws status [7].

This viewpoint article shares strategies on how to detect a forgotten disease such as yaws. My hope is that researchers and dermatologists from the Group B countries, who often are working with minimal resources and no existing yaws programs, may learn from the experience of the Philippines.

## Practical steps to finding yaws

The search for yaws in the Philippines was like finding the proverbial needle in a haystack. Our yaws experts of the 1950s were gone, along with awareness about yaws. Countries without yaws programs do not have a budget for case detection or surveillance, leading to the challenge of how to find a case of active yaws in a country with around 7,100 islands and more than 100 million people.

The strategies below are based on experience from the 2 commissioned yaws studies that I headed as principal investigator and on case detection studies in other countries. These tactics may be useful in assessing for the presence or absence of yaws.

### 1. Search in high-risk areas: Remote villages and among indigenous peoples

Yaws is sporadic with a spotty distribution. WHO suggests that researchers use purposive searches in previously endemic villages, starting with a look at historical records [8] and then a search for remote communities with historically high numbers of reported yaws.

In 2017, we started a search in Liguasan Marsh, where we had first seen yaws 22 years ago. In the second yaws study, we targeted remote villages of indigenous people. In keeping with the success of this approach, in 2021, the first confirmed yaws case in Malaysia since 1985 was a 5-year-old aboriginal child from the Batek tribe [9].

In Liberia, the 15th country designated as endemic for yaws, a different approach was used. Anecdotal reports led researchers to conduct a population-based cluster-randomized cross-sectional survey integrated with other neglected tropical diseases of the skin. This method of detection, however, required a large amount of resources and costs that most Group B countries cannot afford. Timothy and colleagues reported that during the extensive search in 1 district in Liberia, yaws was more likely to be found in remote communities [10].

Boock and colleagues [11] compared 5 methods used for yaws detection in Bankim District, Cameroon, during 2012 to 2015. The methods that yielded >70% of cases were mass outreach programs and school-based screenings in communities where yaws had been detected previously, similar to the strategy in the Philippines. Clinic-based passive detection yielded the least number of cases [11].

### 2. Harness local knowledge

As mentioned, in the year 2000, I identified cases of yaws by chance during a community skin survey in Liguasan Marsh, Maguindanao. Despite being a dermatologist, I did not know at the time what yaws looked like until the local health workers referred these patients to me. “Rumor” reports and rumor registries have been successfully used for yaws campaigns in endemic countries of Africa and in India [12].

### 3. Teach people what yaws looks like

In a country where even dermatologists and workers in the health sector in general no longer recognize yaws, information must be revived and awareness generated about the appearance of yaws, first targeting dermatologists and health personnel and then the general public in villages at high risk for yaws.

In the Philippines, the WHO’s Yaws Recognition Booklet for Communities [13] was translated into various local languages, and photographs of Filipino children with yaws were added to enable easier recognition. In 2017, our index case in Liguasan Marsh was brought in by a midwife who had seen photos during the study orientation.

In the 2017 Philippine yaws study, students were instructed to screen their household members for any skin disease with the help of flyers bearing colored images of yaws and leprosy [5]. Similarly, in Cameroon, students also were taught how to recognize yaws [11], and in Liberia, community health workers showed colored photographs of neglected tropical diseases of the skin, including yaws, to households [10].

### 4. Crowdsource for information on yaws

To reduce research expenses and expedite searches, I decided to crowdsource [14] information on yaws, while also conducting a review of historical records. Study staff emailed the Yaws Recognition Booklet together with a Google form questionnaire to various health professionals and invited them to participate in an online survey asking about their encounters with yaws. Through these online reports, some study sites could be selected without the expense of travel in the search for yaws.

### 5. Set up active surveillance

Health personnel who participated in the online survey also were asked to report any suspected yaws cases to the investigators during the study period (active surveillance). During the second yaws study in 2020, two doctors reported seeing yaws among indigenous peoples in the Luzon island group [15]. These reports may have been few in number but were considered good leads for study sites where case detection surveys would be worthwhile.

### 6. Use community skin health as an entry point for detecting yaws

A community skin health approach can be the most strategic approach because it raises skin health awareness generally without singling out yaws and encourages check-ups for any skin problem during school-based and community clinics. Serologic confirmation of yaws should be ensured in suspected cases. We adopted a community engagement strategy that had proved effective in detecting cases of leprosy in various parts of the country [16].

A similar integrated approach was used effectively in Liberia, prioritizing 4 neglected tropical diseases of the skin: yaws, leprosy, Buruli ulcer, and lymphatic filariasis [17].

**Fig 2:**
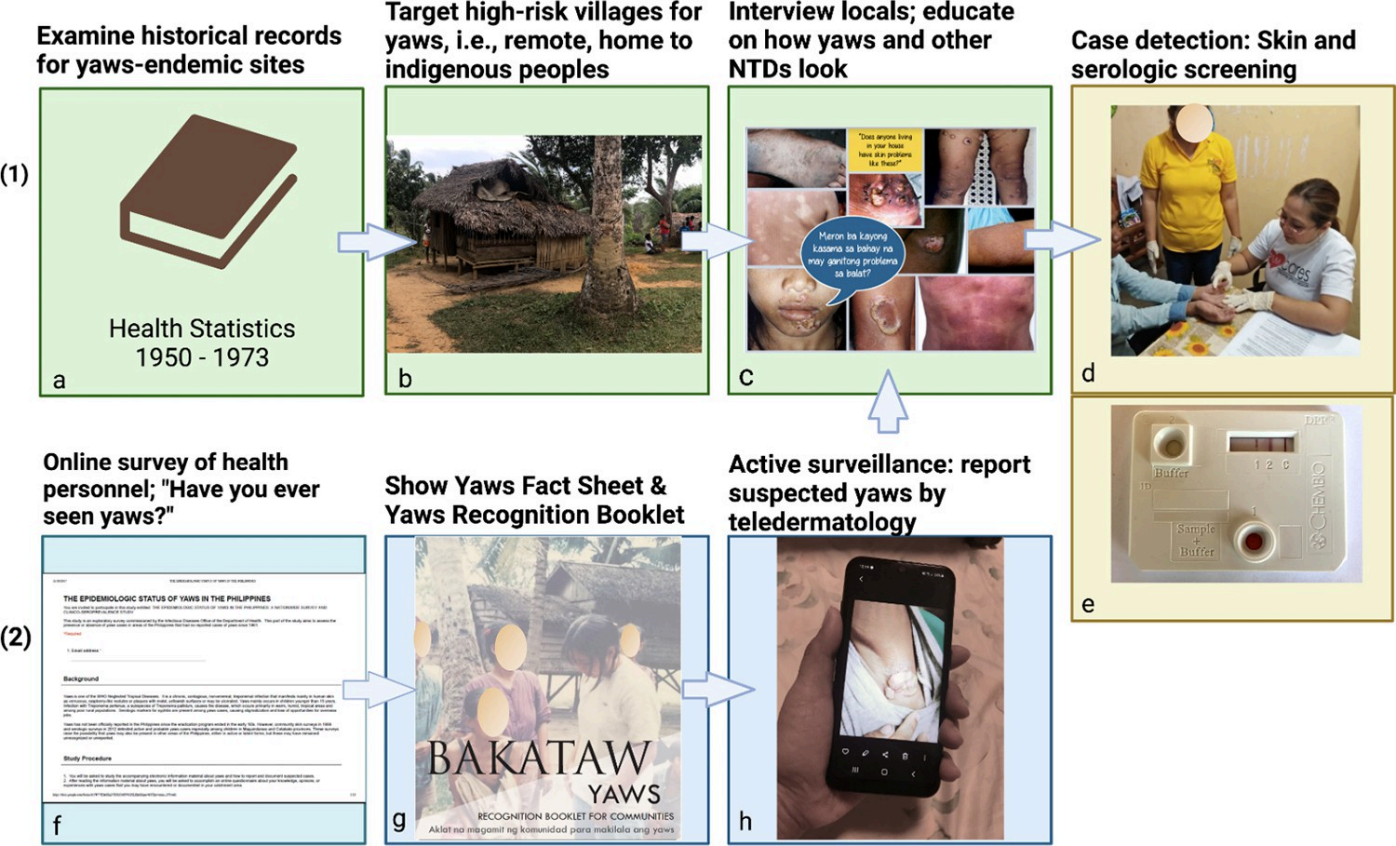
Two-pronged approach to find yaws in a previously endemic country: (1) start with historical records to find yaws-endemic sites and (2) crowdsourcing from health personnel online; show yaws skin signs and ask for any reports or records. (Created with BioRender.com). b. Aeta village, Tagkawayan, Quezon*; c. Flyer of yaws and leprosy photos used during the yaws studies**; NTD Neglected Tropical Diseases; d. Skin examination by dermatologist Dr Belen Dofitas (Sultan Kudarat) 2017*; e. DPP kit*; f. Online survey form used during yaws studies*; g. Cover of Yaws Recognition Booklet for Communities (Filipino version)**; h. Yaws image on a cellphone* *Photo credit Dr. Belen L Dofitas; ** Yaws Recognition Booklet for Communities (Filipino version).

In summary, a two-pronged approach can be used to identify yaws in a previously endemic country where it has been forgotten and status is unknown. Health researchers can start with records to select historically yaws-endemic sites that are remote, underserved, and home to indigenous peoples, interview local health workers, show photographs of yaws, and conduct case detection with skin and serologic screening on children and others with suspected yaws. Simultaneously, crowdsourcing can be conducted to gain information from health personnel nationwide using online methods to raise awareness, educate about yaws skin and bone signs, and request reports or records. This approach also initiates an informal active surveillance that enables health personnel to report suspected yaws cases via teledermatology. If suspected yaws is reported, resources can be focused on the identified village for case detection activities (Fig 2).

## Conclusion

We can detect yaws with strategies focused on communities previously endemic or at high risk for yaws, such as remote and impoverished villages and those that are home to indigenous peoples. Yaws may seem to be a relatively rare disease in the Philippines, but it is not recognized clinically and thus is underreported. If yaws is to be eradicated worldwide, countries with unknown yaws status must first recognize relevant skin and bone signs and resume active surveillance and case detection. With more government support and partnerships with the various sectors, we will be able to make yaws “forgotten no more.”

**Key Learning Points**

- Yaws is a skin neglected tropical disease earmarked for eradication by the WHO, however, around 76 previously endemic countries are not yet assessing yaws status.
- The suggested strategies are based on the experience of finding yaws in the Philippines and in other countries.
- Since yaws has been forgotten and unrecognized in many countries, a two-pronged approach is suggested for case detection: start the search in historically yaws-endemic sites that are remote and home to indigenous peoples, interview local health workers, show photographs of yaws, and conduct skin and serologic screening on children.
- Simultaneously, crowdsource for information online about yaws encounters from health personnel nationwide, raise their awareness about yaws, initiate informal active surveillance, and encourage referrals of suspected yaws cases via teledermatology.
- If yaws is to be eradicated worldwide, countries with unknown yaws status must first recognize relevant skin and bone signs, resume active surveillance, and case detection.

**Top Five Papers**

- Yaws: World Health Organization; 2023 [updated 12 January 2023; cited 2023 January 17, 2023]. Available from: https://www.who.int/news-room/fact-sheets/detail/yaws.
- WHO. Eradication of Yaws—A Guide for Programme Managers. Geneva: World Health Organization; 2018 January 2018. 40 p.
- Dofitas BL, Kalim SP, Toledo CB, Richardus JH. Yaws in the Philippines: A clinico-seroprevalence study of selected communities in Mindanao. PLoS Negl Trop Dis. 2022;16(6):e0010447. doi: 10.1371/journal.pntd.0010447.
- Dofitas B, Batac MC, Richardus JH. Finding Yaws among Indigenous People: Lessons from Case Detection Surveys in Luzon and Visayas Island Groups of the Philippines. Am J Trop Med Hyg. 2022. Epub 20221226. doi: 10.4269/ajtmh.22-0566. PubMed PMID: 36572006.
- Boock A, Awah P, Mou F, Nichter M. Yaws resurgence in Bankim, Cameroon: The relative effectiveness of different means of detection in rural communities. PLoS Negl Trop Dis. 2017;11(5):e00005557.
